# Sexual knowledge, risk behavior, and access to reproductive health services among orphaned adolescents in Southwest Nigeria: implications for institutionalized care

**DOI:** 10.3389/fgwh.2023.1151099

**Published:** 2023-05-16

**Authors:** Ifedola Olabisi Faramade, Adenike Iyanuoluwa Olugbenga-Bello, Olayinka Olufisayo Goodman

**Affiliations:** ^1^ Department of Community Medicine, College of Health Sciences, Osun State University, Oshogbo, Nigeria; ^2^ Department of Community Medicine, College of Health Sciences, Ladoke Akintola University of Technology, Ogbomosho, Nigeria; ^3^ Department of Community Health and Primary Health Care, Lagos State University College of Medicine, Ikeja, Nigeria

**Keywords:** sexual knowledge, risk behavior, access, orphaned adolescents, institutionalized care, reproductive health services, Nigeria

## Abstract

**Introduction:**

An orphan has been defined as a child under 18 years of age who has lost one or both parents to any cause. It has been reported that for every 10 Nigerian children, 1 is likely to be an orphan. Adolescents are faced with a serious challenge in meeting their reproductive health need, which oftentimes becomes overwhelming especially when they are orphaned.

**Objectives:**

We compared institutionalized and non-institutionalized orphaned adolescents for their knowledge of sexuality, risky sexual practice, and access to reproductive health services.

**Methods:**

The study adopted a cross-sectional descriptive study design conducted via structured, pretested, and interviewer-administered questionnaires among 205 orphaned adolescents (140 institutionalized and 65 non-institutionalized). Data were analyzed using the Statistical Product and Service Solution (SPSS version 25.0) and summarized using frequency, mean and percentages, and inferential statistics. All analyses were done at a 95% confidence interval and at a *p* < 0.05 level of significance.

**Results:**

The knowledge levels of a majority of non-institutionalized respondents (73.8%) were good when compared with those in institutions (56.4%) (*χ*^2^ = 5.713, *p* = 0.017). Institutionalized orphans displayed better sexual behavior (80.7%) than non-institutionalized respondents (64.6%) (*χ*^2^ = 6.239, *p* = 0.011). Access to reproductive health services was found to be slightly higher among institutionalized respondents (66.4%) than among their non-institutionalized counterparts (64.6%).

**Conclusion:**

Institutionalized and non-institutionalized orphans differed in terms of their knowledge of sexuality, sexual behavior, and risky practices, including access to reproductive health services. This study demonstrated the effectiveness of institutionalized care of orphans toward improved access to reproductive health services and good sexual practices. In the light of this, the government and relevant stakeholders should advocate the need for providing better sexuality education and understanding, make sure that access barriers for orphans are removed and orphans utilize the facilities for reproductive health that are available, and also make sure that adolescent health policies are implemented effectively.

## Introduction

An orphan has been defined as a child under 18 years of age who has lost one or both parents to any cause (1). Children are orphaned because of several reasons and this exposes them to multiple challenges that include child abuse, child labor, trafficking, malnutrition, HIV/AIDS and other transmissions, declining school enrolments, and lack of educational opportunities (2).

There are an estimated 153 million orphans worldwide, including paternal, maternal, or double orphans (3). In 2015, the Nigeria's Federal Ministry of Women Affairs and Social Development estimated that approximately 17.5 million orphans were vulnerable to various risk factors that included AIDS, unfavorable ethnoreligious and cultural practices, poverty, maternal mortality, sectarian and ethnic conflicts, and gender inequality (4, 5). An analysis of orphaned and vulnerable children done in 2008 gave an estimated figure of approximately 17.5 million in Nigeria (6).

It has been reported that for every 10 Nigeria children, 1 is likely to be an orphan (6). Similarly, while one-third of these orphans are likely to be maternal orphans, the remaining two-thirds are either paternal orphans or both (7, 8).

As described by the World Health Organization (WHO), an adolescent is any person whose age ranges between 10 and 19. Puberty is intimately tied to adolescence and most adolescents manifest risk-taking tendencies during this stage of development (9). Worldwide, adolescents are faced with a serious challenge in meeting their reproductive health need, which oftentimes becomes overwhelming especially when they are orphaned (9).

The state of orphanhood impacts adolescents negatively with its attendant consequences. Most orphans are either socially excluded or lack basic economic support and become prone to various developmental, psychological, social, and health challenges. Beyond an inadequate knowledge of sexuality and risky sexual behaviors, they encounter restricted access to reproductive health experiences and are left with unmet emotional and sexual cognitive needs (10).

Studies have shown that adolescents are one of the most ignored populations in the context of sexual and reproductive health (SRH) due to a combination of two factors: sensitivity of the topic and young age characteristic. Their knowledge about puberty, sexuality, and use of reproductive health services is also less; thus, the ability to use such services will determine the extent to which they will deal with their reproductive health problems (11, 12).

When one parent dies, many orphans remain with the other parent. Paternal orphans oftentimes remain with their mothers, but maternal orphans remain with their fathers in only a few instances and only under certain circumstances. The other existing options of care for orphaned children include institutional care, for example, “orphanages,” while some children are cared for in the community itself by means of traditional kinship or extended families, which is common in the case of double orphans (13). Studies also reported that several orphans preferred residential care to foster care because of varying reasons of abuse and family-distress situations (14).

Freidus described the positive outcomes of institutionalized care as removing children from abusive homes and meeting their physical and educational needs (15). Recently, there has been a gradual increase in the number of orphans requiring support and care. Willing caregivers or family members are faced with overwhelming challenges ranging from poverty to a large household size, with the resultant inability to provide basic needs and resources to support orphaned children (13–15).

The United Nations Convention on the Rights of the Child highlighted the basic human rights of children to include the right to survival, to develop to the fullest to gain protection from harmful influences, abuses, and exploitation, and to participate fully in family, cultural, and social life. The convention fully protects children's rights through a set of standards in healthcare. However, the fact that being an orphan may confer a certain level of disadvantage on the person often seems to be more pronounced among those with peculiarities associated with adolescence and orphanhood (13, 16, 17).

Most studies in developing countries have shown that such adolescents lack some basic rights. A significant proportion of adolescents lack adequate knowledge about reproductive health, principally contraception, and thus, they hold certain misconceptions about their sexuality and reproductive health needs (18). The adolescent fertility rate in Nigeria (births per 1,000 women in the age range of 15–19), which was 102% in 2020, highlights the importance of focusing on adolescents, but factors such as orphanhood, a lack of parental care and so on place barriers on such efforts (19, 20).

Despite the wealth of material available on the enormous challenges faced by orphaned adolescents, there is a dearth of knowledge on their sexual risk behavior and reproductive health services, particularly in Southwestern Nigeria. Against this background, this study was conducted to determine the level of knowledge on sexuality, sexual practices, and access to reproductive health services among institutionalized and non-institutionalized adolescent orphans.

## Methods

### Study design and participants

This study adopted a cross-sectional descriptive study design formulated through structured, pretested, and interviewer-administered questionnaires. The study was conducted in Ibadan, the capital of Oyo State, located in Southwestern Nigeria, which is also the largest indigenous city in Africa. It is situated 78 miles inland from Lagos, and is a prominent transit point between the coastal region and the areas to the north. The principal inhabitants of the city are the Yorubas. It has a population estimated to be approximately 3,649,023 according to the 2021 census.

The sample size of the study was estimated using the Taro Yamane formula for proportions: *n* = *N*/(1 + *N* × 10^2^), where *n* = sample size, *N* = population under study, and *e* = margin error (a standard value of 0.05 was used). A total of 205 orphaned adolescents who participated in this study were purposively selected across communities with orphaned adolescents. A total of 140 respondents from 11 orphanages were selected using the simple random sampling technique to represent institutionalized orphans, while a total sample size of 65 participants representing non-institutionalized orphans was drawn from select centers of worship (churches and mosques) found within the same communities where orphanages were located within Ibadan.

### Statistical analysis

Completed questionnaires were first reviewed for the presence of any inconsistencies. Data were analyzed using the IBM-SPSS version 25 software for Windows. The data were summarized using descriptive statistics such as frequency distribution and mean and percentages, while inferential statistics using the *χ*^2^ statistical test was used to test for differences in knowledge, sexual practices, and access to reproductive health services between institutionalized and non-institutionalized orphaned adolescents. A multivariate analysis was performed using logistic regression analysis for variables that are significant at the binary level. The level of significance was set at *p* < 0.05 and the confidence interval was set at 95%.

### Ethical consideration

Ethical approval for the research was sought and obtained from the Ethical Review Committee of Ladoke Akintola University of Technology (LAUTECH) Teaching Hospital, Ogbomoso, Oyo State, Nigeria. Also, permission to carry out the study was obtained from the Ministry of Women Affairs, Community Development, Social Welfare and Poverty Alleviation, Ibadan, Oyo State, and the relevant management of selected orphanages and religious bodies.

Informed consent was obtained before recruiting all participants in the study after providing a detailed explanation of the aims and objectives of the research. The right of participants to privacy and anonymity was strictly adhered to by the researchers, and respondents were given full assurance of confidentiality. Those who were not willing to participate had the right to decline being interviewed, while they were assured that they would not suffer any consequence for taking such a decision.

## Results

One hundred and forty institutionalized orphaned adolescents were recruited across orphanages while another sixty-five were recruited through total sampling from non-institutionalized settings. The mean age of respondents was 14.6 + 1.67 and 14.7 + 1.55 among institutionalized and non-institutionalized orphaned adolescents respectively (Table 1).

**Table 1:** Sociodemographic characteristics of respondents by category of care (*N*) = 205 (Ibadan, Nigeria, 2020).

Variable	Category of care		Total	*χ* ^2^	df	*p*-value
	Institutionalized *N* = 140 (%)	Non-institutionalized *N* = 65 (%)				
Gender						
Male	69 (49.3)	27 (41.5)	96 (46.8)	1.070	1	0.301
Female	71 (50.7)	38 (58.5)	109 (53.2)			
Grouped age						
10–14 years	68 (48.6)	28 (43.1)	96 (46.8)	0.538	1	0.463
≥15 years	72 (51.4)	37 (56.9)	109 (53.2)			
Mean age (years)	14.6 (±1.67)	14.7 (±1.55)				
Highest educational level						
Primary	18 (12.8)	5 (7.7)	23 (11.2)	5.333	3	0.149
Secondary	110 (78.6)	48 (73.8)	158 (77.1)			
Tertiary	11 (7.9)	10 (15.4)	21 (10.2)			
No formal education	1 (0.7)	2 (3.1)	3 (1.5)			
Occupational status						
Working class	13 (9.3)	3 (4.6)	16 (7.8)	9.221	3	0.026^*^
Learning a trade	29 (20.7)	14 (21.5)	43 (20.9)			
Vocational institutes	8 (5.7)	12 (18.5)	20 (9.8)			
None	90 (64.3)	36 (55.4)	126 (61.5)			
Religion						
Islam	49 (35.0)	26 (40.0)	75 (36.5)	5.859	3	0.021^*^
Christianity	89 (63.6)	37 (56.9)	126 (61.5)			
Traditional	1 (0.7)	0 (0.0)	1 (0.5)			
Others	1 (0.7)	2 (3.1)	3 (1.5)			
Ethnicity						
Yoruba	87 (62.1)	35 (53.8)	122 (59.5)	1.627	3	0.653
Hausa/Fulani	26 (18.6)	13 (20.0)	39 (19.0)			
Igbo	23 (16.4)	15 (23.1)	38 (18.5)			
Others	4 (2.9)	2 (3.1)	6 (3.0)			

^*^ Statistically significant <0.05.

Majority of respondents (53.2%) were females and 77.1% of respondents completed secondary education. About 61.5% of these respondents are not currently involved in any occupation, there is a statistically significant difference between institutionalized orphans in this category and those not in any institution (*χ*^2^ = 9.221, *p* = 0.026). These respondents are predominantly Yoruba (one of the three largest ethnic groups in Nigeria, concentrated in southwestern part of the country) (59.5%) and majority (61.5%) being Christians shows a statistically significant difference compared to those who practiced other religions. (*χ*^2^ = 5.859, *p* = 0.021) (Table 1).

One hundred and thirteen (68.1%) of institutionalized respondents and 31.9% of non-institutionalized respondents reported ever heard of puberty. Regarding age at onset of puberty, majority (89.3%) opined attainment of puberty is between the ages of 10 and 14 years (68.9%) and (31.1%) among institutionalized and non-institutionalized respondents respectively. Some were able to give characteristics of puberty however a statistically significant difference (*χ*^2^ = 5.975, *p* = 0.052) is shown between respondents from both categories who identified puberty as a period when new feelings of interest and attraction towards opposite sex is seen (Table 2).

**Table 2:** Knowledge on sexuality and puberty by category of care (*N*) = 205 (Ibadan, Nigeria, 2020).

Variable	Institutionalized	Non-institutionalized	Total	*χ* ^2^	df	*p*-value
Ever heard of puberty						
Yes	113 (80.7)	53 (81.5)	166 (80.9)	0.020	1	0.889
No	27 (19.3)	12 (18.5)	39 (19.1)			
Age at onset of puberty						
Starts between 10 and 14 years	126 (90.0)	57 (87.7)	183 (89.3)	1.653	2	0.437
Later than 14 years or earlier	7 (5.0)	2 (3.1)	9 (4.4)			
Not sure	7 (5.0)	6 (9.2)	13 (6.3)			
Puberty is characterized^a^						
Bodily changes and development toward adulthood	132 (67.3)	61 (32.7)	193 (94.1)	1.991	2	0.370
Changes in size of genitalia including growth of pubic hair	135 (68.5)	62 (31.5)	197 (96.1)	0.871	2	0.647
Production of sperm cells in males or egg cells in females	120 (67.0)	58 (89.2)	178 (86.8)	1.172	2	0.556
New feelings of interest and attraction toward the opposite sex	123 (62.3)	61 (93.8)	184 (89.8)	5.975	2	0.052^*^
Masturbation in boys/menses among girls	79 (64.8)	43 (66.2)	122 (59.5)	2.076	2	0.354
Sources of information^a^						
Teachers	123 (68.3)	57 (31.7)	180 (87.8)	5.280	1	0.002^*^
Media (TV, radio, internet, social)	56 (84.8	10 (15.2)	66 (32.2)	12.321	1	0.991
Caregivers	27 (93.1)	2 (6.9)	29 (14.1)	9.603	1	0.973
Sexual partners (boyfriends, girlfriends)	15 (68.2)	7 (31.8)	22 (10.7)	0.085	1	<0.001^*^

^a^ Multiple responses.

^*^ Statistically significant.

The major sources of information about puberty and reproductive health issues among the study groups were teachers (87.8%) and sexual partners (boyfriends/girlfriends), but media (television, radio, internet, and social) (32.2%) and caregivers were found to contribute less, and there was no statistical difference between the study arms. There exists a statistically significant difference between the institutionalized (93.1%) and the non-institutionalized respondents (6.9%) obtaining information from their teachers (*χ*^2^ = 5.280, *p* = 0.002) and sexual partners (*χ*^2^ = 0.085, *p* < 0.001) (Table 2).

A majority of non-institutionalized orphans (60.0%) never had any sex, and nearly half of their institutionalized counterparts (48.6%) also had the same experience of no sex. However, this difference was not statistically significant.

Age at first sexual debut among the majority (77.9%) of institutionalized orphans was less than or at 14 years, compared with the majority of non-institutionalized orphans (51.3%), whose age at first sexual experience was above 14 years. This difference was statistically significant, and it was (*χ*^2^ = 12.611, *p* = 0.002).

A majority of orphans in both settings (institutionalized and non-institutionalized) were found to have had no sexual intercourse in the preceding 12 months to the start of the study period (61.4% and 73.9%, respectively). However, 38.6% of institutionalized orphans had sexual intercourse within the same time duration when compared with 26.1% of non-institutionalized orphans, and the difference was not statistically significant.

The common reasons given by the majority (77.3%) of institutionalized respondents for having sexual intercourse were that they were forced, raped, coerced, or tricked, as compared with the majority (46.9%) of non-institutionalized orphans, who gave reasons such as peer pressure, personal expectation and predilection, or willingness to show love to, or to please their, partners. This difference between the respondents was found to be statistically significant (Table 3).

**Table 3:** Association between sexual practice and by category of care of respondents (*N*) = 205 (Ibadan, Nigeria, 2020).

Variables	Institutionalized	Non-institutionalized	Total	*χ* ^2^	df	*p*-value
Ever had sex						
Yes	68 (48.6)	39 (60.0)	107 (52.2)	2.224	1	0.127
No	72 (51.4)	26 (40.0)	98 (47.8)			
Age at first debut (years) (*n* = 107)						
<14	53 (77.9)	19 (48.7)	72 (67.3)	12.611	2	0.002^*^
>14	15 (22.1)	20 (51.3)	35 (32.7)			
Had sex in the last 12 months						
Yes	54 (38.6)	17 (26.1)	71 (34.6)	4.748	1	0.191
No	86 (61.4)	48 (73.9)	134 (65.4)			
Number of sexual partners						
1	15 (27.8)	7 (21.2)	22 (31.0)	6.519	3	0.259
2	32 (59.2)	8 (47.1)	40 (56.4)			
3	4 (7.4)	0 (0.0)	4 (5.6)			
4	3 (5.6)	2 (11.7)	5 (7.0)			
Reasons for sexual intercourse^a^ (*n* = 107)						
Willingness to show love or please partner	21 (58.3)	15 (41.7)	36 (33.6)	25.771	4	0.007^*^
Forced/raped/coerced/tricked	34 (77.3)	10 (22.7)	44 (41.1)			
Personal interest at losing virginity	3 (30.0)	7 (70.0)	10 (9.3)			
Personal expectation/peer pressure	17 (53.1)	15 (46.9)	32 (29.9)			
Drug effect	2 (100.0)	0 (0.0)	2 (1.9)			
Ever received anything/gift in exchange for sex	*n* = 68	*n* = 39				
Yes	21 (30.9)	22 (56.4)	43 (40.2)	10.846	1	0.004^*^
No	47 (69.1)	17 (43.6)	64 (59.8)			
Engaged in sexual intercourse on first date						
Yes	10 (14.7)	6 (15.4)	16 (13.1)	0.416	1	0.721
No	58 (85.3)	33 (84.6)	91 (85.0)			
Ever engaged in oral sex						
Yes	8 (11.7)	4 (10.3)	12 (11.2)	0.057	1	0.812
No	60 (88.3)	35 (89.7)	95 (88.8)			
Ever engaged in anal sex						
Yes	7 (10.3)	9 (23.1)	16 (14.9)	3.185	1	0.074
No	61 (89.7)	30 (76.9)	91 (85.1)			
Insisting on condom use whenever having sex						
Always	6 (8.8)	3 (7.7)	9 (8.4)	5.432	3	0.031^*^
Most times	1 (1.5)	2 (5.1)	3 (2.8)			
Sometimes	30 (44.1)	4 (10.3)	34 (31.8)			
Never	31 (45.6)	30 (76.9)	61 (57.0)			
Abstinence from sexual intercourse when partner's sex history becomes unknown						
Yes	10 (7.1)	55 (84.6)	65 (31.7)	123.04	1	<0.001^*^
No	130 (92.9)	10 (15.4)	140 (68.3)			

^a^ Multiple responses allowed.

^*^ Statistically significant.

A majority of respondents from the two groups had never received anything in the form of a gift in exchange for sex, when compared with those who have had sexual intercourse as an exchange for a gift, and this difference was statistically significant (*χ*^2^ = 10.846, *p* = 0.004). Also, there was a significant difference between non-institutionalized orphans (56.4%) and institutionalized orphans (30.9%) who had exchanged something for sex. Only 13.1% of the total respondents had ever engaged in sexual intercourse on the first date, while 85.0% did not engage in such an act.

Among institutionalized respondents, only 11.7% and 10.3% had ever engaged in oral sex and anal sex, respectively, compared with 10.3% and 23.1% of respondents who were not in any institutionalized care. This difference was not statistically significant. A higher majority of non-institutionalized orphans (76.9%) never used the condom when having sex compared with 45.6% of institutionalized orphans who belonged to the same category. This difference in rate was statistically significant (*χ*^2^ = 5.432, *p* = 0.031).

A statistically significant majority of non-institutionalized orphans (84.6%) abstained from sexual intercourse when their partner’s sexual history remained unknown, compared with a far higher percentage of institutionalized orphans (92.9%) who did not consider abstinence (*χ*^2^ = 123.04, *p* < 0.001).

A majority of orphans in institutions (63.6%) and 76.9% from non-institutionalized care had access to adolescent-friendly sites in their various communities.

From both settings, these centers/services were predominantly reported to be cited/accessed within the hospitals. With regard to their willingness to visit these sites for reproductive health services, a majority (57.9%) of orphans in institutionalized care showed unwillingness to visit these sites, while 66.2% representing a majority of non-institutionalized orphans were willing to access such sites. This difference was statistically significant at (*χ*^2^ = 10.237, *p* = 0.001).

It was found that among institutionalized respondents who never had any sex, a majority of them (69.1%) used the condom during sexual intercourse in the preceding 12 months to the start of the study period, while only 33.3% of non-institutionalized orphans used the same. A higher proportion (58.9%) of those who did not use any contraceptive methods constituted non-institutionalized respondents, compared with 23.5% of their institutionalized counterparts, although this difference was not statistically significant.

With regard to the ease of access to common contraceptives, a statistically significant difference existed between institutionalized orphans (22.8%) who could easily access contraceptives (especially condom) and non-institutionalized orphans, who constituted a higher percentage (33.8). This difference was statistically significant; *p* = 0.001. A majority of institutionalized orphans (45.7%) said that they were not willing to access reproductive health services because it was too embarrassing for them to do so, and another 38.3% reported gave the reason of unfriendliness of healthcare workers for not accessing such services. Non-institutional orphans also stated that the unfriendliness of healthcare workers and their own embarrassment came in their way of accessing reproductive health services, with the percentage being 40.9 and 36.3, respectively. These reasons produced a statistically significant difference of (*χ*^2^ = 11.499, *p* = 0.021). The least reported reason reported by both groups were the unaffordable cost of contraceptives and the lack of confidentiality in the service provided.

Respondents who claimed to have accessed, or were willing to access, the facilities gave various reasons to do so, which ranged from health education and counseling, provision of contraceptives, and treatment of common sexually transmitted diseases (STDs). A majority of respondents who accessed health facilities for availing treatment of common STDs (65.2%) were institutionalized orphans, while only 34.8% were those from non-institutionalized care. Similarly, a statistically significant difference of (*χ*^2^ = 6.609, *p* = 0.037) existed between the 64.3% of institutionalized respondents whose perceived reasons for accessing or willingness to access reproductive health services/facilities were to address issues around reproductive health issues, health education and counselling compared with 35.7% of non-institutionalized respondents. Likewise, there was a statistically significant difference between those whose reasons are for providing contraceptives (67.6%) and (35.3%) (Table 4).

**Table 4:** Association between access to reproductive health services and orphaned respondents by category of care (*N* = 205) (Ibadan, Nigeria, 2020).

Variables	Category of care of respondents		Total	*χ* ^2^	df	*p*-value
	institutionalized	Non-institutionalized				
Presence of adolescent-friendly sites within the community						
Yes	89 (63.6)	50 (76.9)	139 (67.8)	3.751	2	0.153
No	42 (30.0)	13 (20.0)	55 (26.8)			
Don't know	9 (6.4)	2 (3.1)	11 (5.4)			
Location of sites or center (*n* = 139)						
School	43 (48.3)	31 (62.0)	74 (53.3)	6.498	2	0.093
Hospital	39 (43.8)	15 (30.0)	54 (38.8)			
Religious setting	7 (7.9)	4 (8.0)	11 (7.9)			
Willingness to visit sites within community for RHS						
Yes	59 (42.1)	43 (66.2)	102 (49.7)	10.237	1	0.001^*^
No	81 (57.9)	22 (33.8)	103 (50.3)			
Commonly accessed type of contraceptives/type used during the last sexual intercourse (*n* = 107)						
Condom	47 (69.1)	13 (33.3)	60 (56.1)		2	0.138
Pills	5 (7.4)	3 (7.7)	8 (7.5)	3.960		
None	16 (23.5)	23 (58.9)	39 (36.4)			
Ease of access to common/listed contraceptives						
Very easy	32 (22.8)	8 (12.0)	40 (19.5)	16.869	3	0.001^*^
Indifferent	17 (12.2)	22 (33.8)	39 (19.0)			
Difficult	23 (16.4)	14 (21.5)	37 (18.0)			
Don't know	68 (48.6)	21 (32.3)	89 (43.5)			
Reasons for unwillingness to access RHS (*N* = 103)						
Too embarrassing	37 (45.7)	8 (36.3)	45 (43.9)	11.499	3	0.021^*^
Unfriendliness of healthcare workers	31 (38.3)	9 (40.9)	40 (38.8)			
Cost is unaffordable	8 (9.8)	2 (9.1)	10 (9.7)			
Lack of confidentiality	5 (6.2)	3 (13.7)	8 (7.6)			
Perceived reasons for visiting RHS site/facility^a^						
Addressing adolescent RH issues	101 (64.3)	56 (35.7)	157 (76.6)	6.609	2	0.037^*^
Provision of contraceptive	96 (67.6)	46 (32.4)	142 (69.3)			
Treatment of STDs	103 (65.2)	55 (34.8)	158 (77.1)			

RHS, reproductive health services.

^a^ Multiple responses.

^*^ Statistically Significant.

Table 5 shows the association between summarized scores of knowledge of sexuality, sexual practice, and access to reproductive health services in relation to the categories of care of adolescent orphans. A statistically significant association exists between institutionalized respondents and the non-institutionalized ones in terms of their knowledge of sexuality. A majority of non-institutionalized respondents (73.8%) showed good awareness compared with 56.4% of orphans in institutions, a difference that was statistically significant at (*χ*^2^ = 5.713, *p* = 0.017). Also, the rate (80.7%) with regard to good sexual practices among orphans in institutions was higher than that of their non-institutionalized counterparts, which was 64.6%, (*χ*^2^ = 6.239, *p* = 0.011).

**Table 5:** Association between summarized scores of outcome variables with category of care of respondents (*N* = 205) (Ibadan, Nigeria, 2020).

Variables	Category of care		*χ* ^2^	df	*p*-value
	Institutionalized	Non-institutionalized			
Knowledge of sexuality					
Good	79 (56.4)	48 (73.8)	5.713	1	0.017^*^
Poor	61 (43.6)	17 (26.2)			
Sexual practice					
Good	113 (80.7)	42 (64.6)	6.239	1	0.011^*^
Poor	27 (19.3)	23 (35.4)			
Access to RHS					
Good	93 (66.4)	42 (64.6)	0.065	1	0.459
Poor	47 (33.6)	23 (35.4)			

RHS, reproductive health services.

*Statistically significant <0.05.

The rate of access to reproductive health services was found to be good in the two groups, although it was slightly higher (66.4%) among the institutionalized orphans than among their non-institutionalized counterparts (64.6%), but this difference was not statistically significant.

The multivariate analysis showed that orphans in institutionalized care whose source of information was their teachers were approximately 4 times more likely to have sound knowledge on puberty, sexuality, and reproductive health services than those with other sources of information, whereas among the non-institutionalized orphans, those who depended on their teachers were about twice more likely to have such good awareness (Table 6).

**Table 6:** Logistic regression analysis to determine the association between knowledge of puberty, sexuality, and RHS; sexual practice and access to RHS among respondents by category of care (Ibadan, Nigeria, 2020).

Institutionalized				Non-institutionalized		
Explanatory factors	B	OR (95% CI)	df (*p*-value)	B	OR (95% CI)	df (*p*-value)
Knowledge of puberty, sexuality, and RHS						
Sources of information						
Teachers (Ref)						
Caregivers/Sexual partners (boyfriends, girlfriends)	0.621	3.746 (0.638–4.431)	3 (0.028)^*^	3.828	1.950 (3.611–110.21)	3 (<0.001)
Sexual practice						
Age at first debut (years)						
<14 (ref)						
>14	1.132	1.075 (0.255–4.538)	1 (0.922)	0.912	0.722 (0.178–2.929)	1 (0.648)
Ever received anything/gift in exchange for sex						
Yes (Ref)						
No	1.189	0.992 (0.571–6.954)	1 (0.275)	1.048	1.357 (0.237–7.754)	1 (0.731)
Insists on condom use whenever having sex						
Always/most times (Ref)						
Sometimes/never	1.742	6.200 (0.704–54.614)	3 (0.067)	5.100	11.250 (1.417–89.259)	3 (0.008)
Abstinence from sexual intercourse once partner's sex history is unknown						
Yes (ref)						
No	1.051	1.253 (0.253–6.211)	1 (0.782)	1.117	2.5 (0.345–6.857)	1 (0.572)
Access						
Willingness to visit sites within community for RHS						
Yes (ref)						
No	1.046	1.247 (0.539–2.885)	1 (0.605)	1.057	1.206 (0.394–3.685)	1 (0.743)
Ease of access to common/listed contraceptives						
Very easy (ref)						
Indifferent/difficult	2.584	5.565 (2.617–11.837)	1 (<0.001)*	0.667	0.546 (0.190–1.566)	1 (0.257)
Perceived reasons for visiting RHS site/facility						
Addressing adolescent RH issues (ref)						
Provision of contraceptive/treatment of STDs	0.534	0.054 (0.021–0.137)	1 (<0.001)*	0.906	0.752 (0.385–1.469)	2 (0.403)

* Represents statistical significance since *p*-value is <0.05.

RHS, reproductive health services.

Institutionalized orphans who have always insisted on condom use whenever having sex are shown to be about six times more likely to be those with good sexual practices compared with non-institutionalized orphans in the same category, who are about 11 times more likely to adopt such healthy practices.

Non-institutionalized orphans will abstain from sexual intercourse about three times more than their institutionalized counterparts if their partner’s sexual history is unknown.

Institutionalized orphans who claimed to have easy access to common contraceptives were about six times more likely to have good access to more reproductive health services compared with those who find it difficult to do so, whereas among non-institutionalized orphans, those who can easily gain access to contraceptives were about twice less likely to have access to more reproductive health services. This difference between the two groups was statistically significant at *p* < 0.001.

Among respondents who are in institutions, those whose perceived reasons for accessing reproductive health services are in order to address their reproductive health issues are about 13 times more likely to make good use of and access further reproductive health services compared with those having other reasons to do so and also with their non-institutionalized counterparts.

## Discussion

This study aimed at determining the knowledge of sexuality, sexual behavior and practice, and access to reproductive health services among institutionalized and non-institutionalized orphaned adolescents living in Ibadan, Oyo State, Nigeria.

This sociodemographic study is similar to that of a study on underserved adolescent orphans in Sagamu, Ogun State, where a majority of respondents were females and were at least 15 years of age, but it is at variance with a Zambian study that reported an even distribution of age and sex among samples (21, 22).

This study identified a high level of awareness on sexuality, especially related to puberty, among orphans from the two study groups. A similar finding was reported in a study on adolescents in Ibadan, Oyo State (23).

While non-institutionalized orphans displayed awareness about their bodily changes and the expected physical changes associated with puberty, their peers in institutionalized settings had a better understanding of the appropriate age of onset of puberty and the associated bodily changes that accompany puberty. Therefore, overall, non-institutionalized orphans displayed a good knowledge of sexuality and puberty. This may be linked to the possibility of such orphans obtaining correct information provided in some schools through sex education, as these schools have been found to provide accurate and comprehensive education about sexuality and healthy sexual behaviors. Some of them also have access to teaching aids in such settings, compared with their counterparts, who reside either with their families or in arranged homes where they have no, or restricted, access to such facilities. This was reported as a likely reason for the low awareness of sexuality education among orphans in a study conducted in Kenya, while in another study, sexuality education for children and adolescents provided by schools and other professionals was identified as being important to help children and adolescents make informed, positive, and safe choices (24, 25). The major source of information for institutionalized orphans was their caregivers and media (television, radio, internet, and social), whereas their non-institutionalized counterparts depended predominantly on their teachers in schools and on their sexual partners (boyfriends or girlfriends) for information (25).

Also, an Iranian study reported by Ganji et al. discovered a majority of sexuality-related issues. Hence, parents depended on a school-based strategy for their children's sexual socialization. Teacher's role in children's sexual health promotion is undeniable, and they play an important role in promoting sexual health and formal school-based education above other sources, which can easily be influenced by community or cultural norms (26).

Also, another study conducted in Kenya that reported about adolescent orphans not practicing caregiver–orphan communication of sexual and reproductive health as a result of poor bonding may explain why sufficient knowledge is not imparted through caregivers as a major source of information among institutionalized orphans who identify them as their source of information (24).

A higher percentage of non-institutionalized respondents reported to have had sexual intercourse at above 14 years of age. The age predominantly reported for institutionalized respondents varied; it was either more or less than 14 years or at the same number. Early sexual debut was reported among adolescent orphans in a study conducted in four sub-Saharan African countries. This could be a result of a combined male and female residential arrangement in some orphanages and inadequate facilities in some makeshift living arrangements. This study further illuminates the reasons associated with sexual intercourse from both groups. A majority of sexual behaviors of sexually experienced non-institutionalized orphans were either coercive encounters, exploitation, or forced or transactional sex (25–27). This finding is at variance with what was reported in the Zambian study among orphans and vulnerable children (OVC), where early sexual debut was reported among out-of-school orphans who also engaged in income-generating activities, a separate study of adolescent orphans also found that female double orphans are more likely to experience an early sexual debut than non-orphans (22, 27).

Orphans in family-based settings may be at an increased risk of exchange of transactional sex and other forms of sexual violence such as risky sexual behaviors and unprotected sexual intercourse compared with those in institutions. This is supported by findings drawn from studies on orphaned adolescents in Western Kenya and Addis Ababa (24, 28).

Previous studies have linked premarital sexual intercourse and risky sexual behavior among adolescents to poor parental relationship with their children and family, societal and school environment, culture, tradition, economic condition, peer relationship, love, and affairs (28, 29).

Also, research findings suggest that sexual behaviors and risks are determined by care environments. Living arrangements under institutional care seem to confer a level of independent protection on orphan adolescents, especially relating to transactional sex. Expectedly, orphans who reside in family-based, non-institutionalized settings have a higher risk of being involved in transactional sex compared with their institutionalized peers and will possibly not insist on using contraceptives (especially condom) during sexual intercourse (30, 31). This may also be related to their restricted access to reproductive health services compared with their institutionalized counterparts. Orphans under institutionalized care settings, irrespective of their partners’ sexual history, will not abstain from sexual intercourse possibly because of a reduced level of overall knowledge on puberty, sexuality, and reproductive health services among them compared with that of their counterparts.

Surprisingly, the rate of overall good sexual practices among the institutionalized orphan respondents, which was found to be higher than that of their counterparts despite their having a reduced level of knowledge, could be attributable to various reasons. This study reported on their increased willingness to visit sites where reproductive health services are offered, and a possible reason for this could be their ease of access to contraceptives compared with their non-institutionalized peers. This is similar to a finding from a study conducted by Casey et al. on adolescents and young women in humanitarian settings, which reported a high level of contraceptive knowledge but low usage, which was thus interpreted to mean a high unmet need for contraception (32). In this study, orphans, predominantly in institutionalized settings who showed a higher involvement in sexual practices at a relatively young age and in multiple sexual relationships, identified reasons ranging from being forced, raped, coerced, or tricked to other forms of abuse, and this is a matter of concern (33). This corroborates findings from orphan and vulnerable children who are subjected to sexual coercion and those reported by Fite and Cherie in Ethiopia. Higher chances of engaging in risky sexual behavior could be a result of not having economic freedom or lack of negotiation power to achieve adequate means of daily subsistence, poor physical, psychological, or emotional protection from caregivers, limited resources, and poor connectedness or monitoring (28).

Conversely, orphans in non-institutionalized care environment engage more in transactional or exchanged sex than those in the other category. This finding has implications for risky sexual behavior and exploitation, although it differs between orphaned adolescents in institutional care settings and non-institutional or family-based environments. Policies and additional support for orphan adolescents in these settings are therefore strongly advocated. The effect of the care environment contributes to differences in sexual behavior and the overall wellbeing of affected orphans (13, 34).

There was a strong correlation between respondents' willingness to visit a site where reproductive health services (RHS) services are provided, ease of access to commonly used modern contraceptives, reasons for willingness to access them, perceived reasons for visiting RH facilities, and the orphan care category. Thus, the findings of this study have implications for future policies and programs, and focused interventions particularly targeted at orphans vulnerable to HIV/AIDS and sexually transmitted infections are called for.

## Conclusion

This study highlights gaps in reproductive health knowledge on sexuality and puberty, access to services, and sexual practices between institutionalized and non-institutionalized orphans. It reveals that the category of care mostly determines orphans’ knowledge of sexuality and access to reproductive health services and contraceptives. The study demonstrates the effectiveness of institutionalized care of orphans toward improved access to reproductive health services and good sexual practices. The knowledge of the above aspects was higher among non-institutionalized orphans than among institutionalized respondents, but this did not translate to the practice of healthy sexual relationship among them.

## Data Availability

The original contributions presented in the study are included in the article, and further inquiries can be directed to the corresponding author.
